# Gastric Antimicrobial Peptides Fail to Eradicate *Helicobacter pylori* Infection Due to Selective Induction and Resistance

**DOI:** 10.1371/journal.pone.0073867

**Published:** 2013-09-11

**Authors:** Sabine Nuding, Michael Gersemann, Yoshio Hosaka, Sabrina Konietzny, Christian Schaefer, Julia Beisner, Bjoern O. Schroeder, Maureen J. Ostaff, Katunori Saigenji, German Ott, Martin Schaller, Eduard F. Stange, Jan Wehkamp

**Affiliations:** 1 Dr. Margarete Fischer-Bosch-Institute of Clinical Pharmacology and University of Tübingen, Stuttgart, Germany; 2 Department of Internal Medicine I, Robert-Bosch Hospital, Stuttgart, Germany; 3 Department of Internal Medicine, Isuzu Hospital, Tokyo, Japan; 4 Department of Gastroenterology, Kitasato University, Kanagawa, Japan; 5 Department of Clinical Pathology, Robert-Bosch Hospital, Stuttgart, Germany; 6 Department of Dermatology, Eberhard Karls University, Tübingen, Tübingen, Germany; University of Hyderabad,, India

## Abstract

**Background:**

Although antimicrobial peptides protect mucus and mucosa from bacteria, *Helicobacter pylori* is able to colonize the gastric mucus. To clarify in which extend Helicobacter escapes the antimicrobial defense, we systematically assessed susceptibility and expression levels of different antimicrobial host factors in gastric mucosa with and without *H. pylori* infection.

**Materials and Methods:**

We investigated the expression levels of HBD1 (gene name *DEFB1*), HBD2 (*DEFB4A*), HBD3 (*DEFB103A*), HBD4 (*DEFB104A*), LL37 (*CAMP*) and elafin (*PI3*) by real time PCR in gastric biopsy samples in a total of 20 controls versus 12 patients colonized with *H. pylori*. Immunostaining was performed for HBD2 and HBD3. We assessed antimicrobial susceptibility by flow cytometry, growth on blood agar, radial diffusion assay and electron microscopy.

**Results:**

*H. pylori* infection was associated with increased gastric levels of the inducible defensin HBD2 and of the antiprotease elafin, whereas the expression levels of the constitutive defensin HBD1, inducible HBD3 and LL37 remained unchanged. HBD4 was not expressed in significant levels in gastric mucosa. *H. pylori* strains were resistant to the defensins HBD1 as well as to elafin, and strain specific minimally susceptible to HBD2, whereas HBD3 and LL37 killed all *H. pylori* strains effectively. We demonstrated the binding of HBD2 and LL37 on the surface of *H. pylori* cells. Comparing the antibacterial activity of extracts from *H. pylori* negative and positive biopsies, we found only a minimal killing against *H. pylori* that was not increased by the induction of HBD2 in *H. pylori* positive samples.

**Conclusion:**

These data support the hypothesis that gastric *H. pylori* evades the host defense shield to allow colonization.

## Introduction

Using 16S rRNA sequencing a great diversity of gastric microorganisms has been shown in the stomach [1]. A protecting mucus layer, the low pH of gastric juice and antimicrobial peptides help to avoid colonization of the gastric epithelium by pathogens with *Helicobacter pylori* being a prominent exception leading to persistent infection and chronic active gastritis [2,3]. *H. pylori* migrates along the pH gradient in the mucus layer and colonizes a niche in the mucus close to the epithelium. About half of the world’s population is colonized. Although most infections remain asymptomatic, *H. pylori* is a risk factor for gastritis and a major cause for gastric and duodenal ulcers [3–5]. Furthermore *H. pylori* colonization is associated with adenocarcinoma of the stomach and gastric MALT lymphoma [6].

Normally, a protective barrier of numerous antimicrobial peptides keeps the bacterial population bordering the gastrointestinal mucus and epithelium under control [7–10]. One important group of these mucosal antimicrobials are defensins, which are cationic peptides with low molecular weight (3-6 kDa) [11]. They are synthesized constitutively or inducible by microbial components or inflammatory stimuli. Defensins exert a broad activity against Gram-positive and Gram-negative bacteria, yeasts, fungi and viruses. The common mode of action of antimicrobial peptides includes the initial electrostatical binding to the negatively charged microbial cell membrane and subsequent incorporation into the membrane. This can lead to an impaired function or even disruption of the cell membrane by pore formation. Aside from defensins, epithelial cells generate the amphipathic α-helical cathelicidin LL37, which also exhibits a wide spectrum antimicrobial activity. Similar to defensins another epithelial peptide, the antiprotease elafin, also has low molecular weight, cationic charge and 4 disulphide bridges, as well as antimicrobial activity [12–15].

*H. pylori* is known to induce the mucosal human beta defensin 2 (HBD2) [16–18] while other studies showed an overexpression of HBD3 [19] and LL37 [20]. Investigations focusing on the antimicrobial activity of these innate immune factors with respect to *H. pylori* [20,21] are limited and it remains unclear whether and how this bacterium evades the attack by these various antimicrobial factors. In the present study, we therefore systematically tested the pattern of induction of antimicrobial peptides by *H. pylori* as well as its susceptibility to the same peptides.

## Methods

### Ethics Statement

All patients gave their written informed consent and the study was approved by the ethical committee of the University of Tübingen, Germany.

### Patients

In a total of 32 patients, who underwent routine gastroscopy, biopsies from the gastric corpus and antrum were collected at the Department of Gastroenterology, Robert Bosch Hospital, Stuttgart, Germany.

Twelve of these patients had a *H. pylori* positive gastritis (mean of age 60 (range 33-81); 50% female) and 20 were *H. pylori* negative controls (mean of age 42 (range 23-61); 35% female). In each patient, one biopsy from the antrum was immediately formalin fixed and sent to the Department of Pathology, Robert Bosch Hospital, for histological evaluation. These biopsies were also immunostained for HBD2 and HBD3. In one biopsy out of the corpus and in one biopsy out of the antrum the urease activity was measured in each patient. Further biopsies were immediately snap-frozen in liquid nitrogen. They were used for RNA and protein extraction and subsequent real-time PCR experiments or flow cytometric assays.

### Histology and urease testing

Antrum biopsies used for histological evaluation were formalin fixed, paraffin embedded and stained with haematoxylin and eosin and assessed according to the Sydney classification [22]. The grade of inflammation was evaluated in these slides by an experienced pathologist of the Department of Pathology. *H. pylori* status was assessed in parallel by methylene blue staining. Urease activity was measured immediately after gastroscopy in the antrum and corpus biopsies with the HUT-test (*H. pylori* Urease-Test, AstraZeneca, Wedel, Germany) according to the supplier’s protocol. The *H. pylori* status of each patient was considered to be positive if *H. pylori* was detected by methylene blue staining and/or by the Helicobacter-Urease-test.

### RNA isolation and reverse transcription

Snap frozen biopsies were mechanically shredded and total RNA was isolated using TRIzol reagent (Invitrogen, Carlsbad, CA, USA) as described previously [23]. RNA quality was verified with the Agilent RNA 6000 Nano Kit (Agilent Technologies, Santa Clara, CA, USA). 500 ng of total RNA was reverse transcribed with AMV reverse transcriptase according to the supplier’s protocol (Promega, Mannheim, Germany). Real-time PCR measurements were carried out with these cDNA preparations.

### Quantitative real-time PCR

Quantitative real-time PCR was performed for β-actin, IL8, HBD1-4, elafin and LL37 in a SYBR Green fluorescence temperature cycler (LightCycler^®^, Roche Diagnostics, Mannheim, Germany) as described previously [23,24]. Single-stranded cDNA (or gene-specific plasmids as controls) corresponding to 10 ng of RNA was used as a template with specific oligonucleotide primer pairs (Table 1). Primers were checked for specific binding to the sequence of interest using BLAST. Plasmids for each product were synthesized with the TOPO TA Cloning Kit (Invitrogen, Carlsbad) as proposed by the supplier. PCR-amplified DNA fragments were sequenced to confirm the correctness of the inserts. Internal standard curves were produced by serial dilution of the plasmids. The mRNA data were normalized to β-actin mRNA.

**Table 1:** Oligonucleotide primer pairs used for PCR measurements.

**Product**	**Forward primer (5´-> 3´**)	**Reverse primer (5´-> 3´**)
**β-actin**	GCCAACCGCGAGAAGATGA	CATCACGATGCCAGTGGTA
**IL8**	ATGACTTCCAAGCTGGCCGTGGC	TCTCAGCCCTCTTCAAAAACTTC
**HBD1** (*DEFB1*)	TTGTCTGAGATGGCCTCAGGTGGTAAC	ATACTTCAAAAGCAATTTTCCTTTAT
**HBD2** (*DEFB4A*)	ATCAGCCATCAGGGTCTTGT	GAGACCACAGGTGCCAATTT
**HBD3** (DEFB103A)	TGAAGCCTAGCAGCTATGAGGATC	CCGCCTCTGACTCTGCAATAA
**HBD4** (*DEFB104A*)	AGATCTTCCAGTGAGAAGCGA	GACATTTCTTCCGGCAACGG
**Elafin** (*PI3*)	CGTGGTGGTGTTCCTCATC	TTCAAGCAGCGGTTAGGG
**LL37** (*CAMP*)	TCGGATGCTAACCTCTACCG	GGGTCACTGTCCCCATACAC

### Immunohistochemistry

HBD2 and HBD3 were immunostained in 5 *H. pylori* negative and 5 *H. pylori* positive antrum biopsies, as well as in 1 Crohn’s colitis and 1 unspecific bronchiolitis sample as positive controls with a two-step immunoperoxidase technique (EnVision^TM^, Dako, Glostrup, Denmark) as described previously [25]. Antigen retrieval was carried out by heating the slides 30 minutes in a steamer in Target Retrieval Solution (Dako, Glostrup, Denmark; pH 9 for HBD2 and pH 6 for HBD3). Then, sections were incubated for 1 hour with the primary anti-HBD2 (Peptide Institute, Osaka, Japan) and anti-HBD3 antibody (Santa Cruz, CA, USA). Anti-HBD2 was diluted 1:300 and anti-HBD3 1:50 in TBST (20mM Tris-Base (pH 7.4), 0.14M NaCl, 0.1% Tween 20). Visualization was performed with a detection kit as outlined by the supplier (Dako: horse-radish-peroxidase (HRP)-labeled secondary antibody, detection with 3'-diaminobenzidine tetrahydrochloride). Sections were counterstained with hematoxylin.

### Bacterial strains and growth conditions

The clinical isolate *H. pylori* KaE1 and the reference strains *H. pylori* DSM 9691, DSM 10242 and DSM 21031 were tested for susceptibility towards defensins, LL37 and elafin (amino acid sequences Table S1). The strain *H. pylori* DSM 10242 is positive for the virulence factors VacA and CagA. As control strain for antimicrobial activity served the sensitive *E. coli* ATCC 25922.

*H. pylori* strains were cultured on Columbia blood agar at 37 C° for 2 days in a microaerophilic atmosphere with CampyGen (Oxoid, Hampshire, UK). Subsequently the bacteria were suspended in Schaedler Broth (BD, Sparks, USA) diluted 1:6 with aqua dest. for incubation with antimicrobial peptides.

### Antimicrobial assays and transmission electron microscopy

For testing antimicrobial activity protein from 5 *H. pylori*-negative and 5 *H. pylori*-positive antrum biopsies was extracted as described [26]. Briefly, the biopsies were pulverized in liquid nitrogen and acid soluble proteins were extracted with 5% acetic acid under agitation for 2 hours with addition of protease inhibitors (PMSF 0.02 mM, pepstatin 2 µg/ml, leupeptin 2 µg/ml). The proteins in the supernatant were dried under vacuum and resuspended in 0.01% acetic acid.

Bacterial viability after incubation with antimicrobial peptides or biopsy extracts was analyzed with the membrane potential sensitive dye DiBAC_4_(3) (bis-(1,3-dibutylbarbituric acid) trimethine oxonol) (Invitrogen, Carlsbad, CA, USA) as described previously [26]. Briefly, 1.5x10^6^ mid-logarithmic-phase bacteria/ml of *H. pylori* or *E. coli* in Schaedler Broth 1:6 were incubated with 20 µg/ml HBD1-4 (Peptide Institute, Osaka, Japan), elafin (Proteo Biotech, Kiel, Germany for FACS analysis and RDA and elafin from Peptide Institute, Osaka for CFU determination), with LL37 (Innovagen, Lund, Sweden) or with 10 µg total protein of antrum biopsy extracts at 37°C. After 90 minutes 1 µg/ml DiBAC_4_(3) was added to the suspensions and further incubated for 10 minutes. Then, the suspensions were centrifuged with 4500 x *g* for 10 minutes, pellets resuspended in 300 µl PBS and bacterial fluorescence was analyzed by a FACSCalibur^TM^ (BD, Sparks, USA), using Cell Quest software (BD, Sparks, USA). In each sample 10000 bacterial events were analyzed for green fluorescence and the antimicrobial activity was determined as percentage of depolarized, fluorescent bacteria. Inhibition of bacterial growth was tested by plating aliquots on Columbia blood agar and incubating at 37 C° for 2 days in a microaerophilic atmosphere with CampyGen (Oxoid, Hampshire, UK). Then colony forming units (CFU) were counted.

Additionally to determine inhibition of bacterial growth we performed radial diffusion assays with a modified protocol of the procedure from Robert Lehrer et al. [27]. Briefly, *H. pylori* was grown on Columbia blood agar (BD, Sparks, USA), suspended in Brain Heart Infusion diluted 1:5 with aqua dest. and plated on an agar containing 20% brain heart infusion with 5% fetal calf serum. Wells with 3 mm diameter were punched into the agar and peptides were applied equivalent to the flow cytometric assay in a concentration of 20 µg per well. The agar plates were incubated for 3 days at 37 °C in a microaerophilic atmosphere with CampyGen.

For electron microscopy 1.5x10^8^ bacteria were incubated with 200 µg peptide/ml for 2 hours. Subsequently bacteria were fixed with Karnovsky solution, embedded in agarose, coagulated, cut in blocks and fixed once more in Karnovsky. After post fixation and embedding in glycid ether the specimen were cut with an ultra microtome. Sections of 30 nm were rinsed on copper grids and investigated with a Zeiss LIBRA 120 transmission electron microscope.

### Binding of antimicrobial peptides to bacterial cells

To investigate whether HBD2 and LL37 bind to the bacterial cell membrane, we incubated *E. coli* or *H. pylori* in Schaedler Broth 1:6 with 20 µg/ml biotinylated LL37 (Innovagen, Lund, Sweden) or 20 µg/ml HBD2 (Peptide Institute, Osaka, Japan) for 30 minutes. After centrifugation a biotinylated HBD2-antibody was added to the in PBS resuspended pellet of the HBD2 samples (Peprotech, Hamburg, Germany). Following washing with PBS, binding of biotinylated LL37 or of the HBD2-antibody was detected with Streptavidin-PE (BD Biosciences, Heidelberg, Germany). Suspensions incubated with HBD2 antibody and Streptavidin served as control. Fluorescence 2 of 10 000 events was measured with the FACSCalibur.

### Statistics

All statistical analyses and graphs were carried out using Prism 5.0 software. Data are presented as means with standard error of the mean (SEM). For comparison of non parametric quantitative real time PCR data the Mann-Whitney test was used. The Spearman’s rank analysis was performed for nonparametric correlation between the different groups. Values of p< 0.05 were considered statistically significant.

## Results

### Expression of HBD1-4, elafin and LL37 in relation to the *H. pylori* status

**Figure 1:**
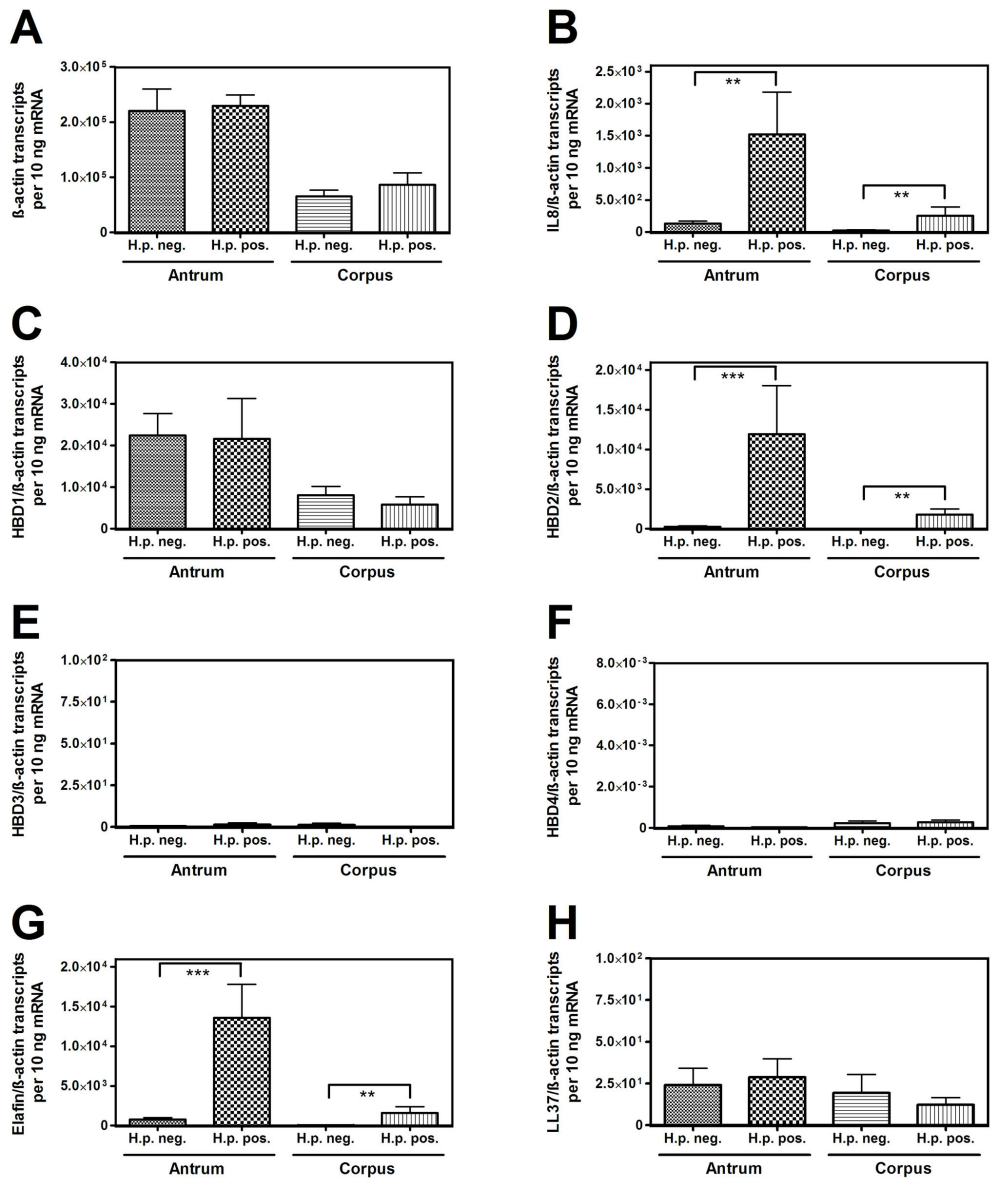
β-actin, IL8, HBD1-4, elafin and LL37 expression in relation to *H. pylori*. β-actin (A) expression is almost equal in *H. pylori* negative and positive antrum and corpus. IL8 (B) transcripts are comparably induced in *H. pylori* antrum and corpus gastritis. HBD1 (C) is constitutively expressed, whereas HBD2 (D) and elafin (G) are significantly enhanced in *H. pylori* populated tissue. HBD3 (E), HBD4 (F) and LL37 (H) mRNA was only marginally detected (**: p<0.01, ***: p<0.001).

*H. pylori* was negative in 20 patients, whereas 12 patients had a positive urease test. Histologically all *H. pylori* positive biopsies showed a moderate chronic active gastritis, whereas in the *H. pylori* negative specimens no or little chronic inflammation occurred. Beta-actin transcripts were comparable in *H. pylori* positive and negative subgroups in both antrum and corpus (Figure 1A). IL8 mRNA (Figure 1B) was significantly induced in *H. pylori* positive samples (antrum: 12-fold, p=0.004; corpus: 11-fold, p=0.002).

**Figure 2:**
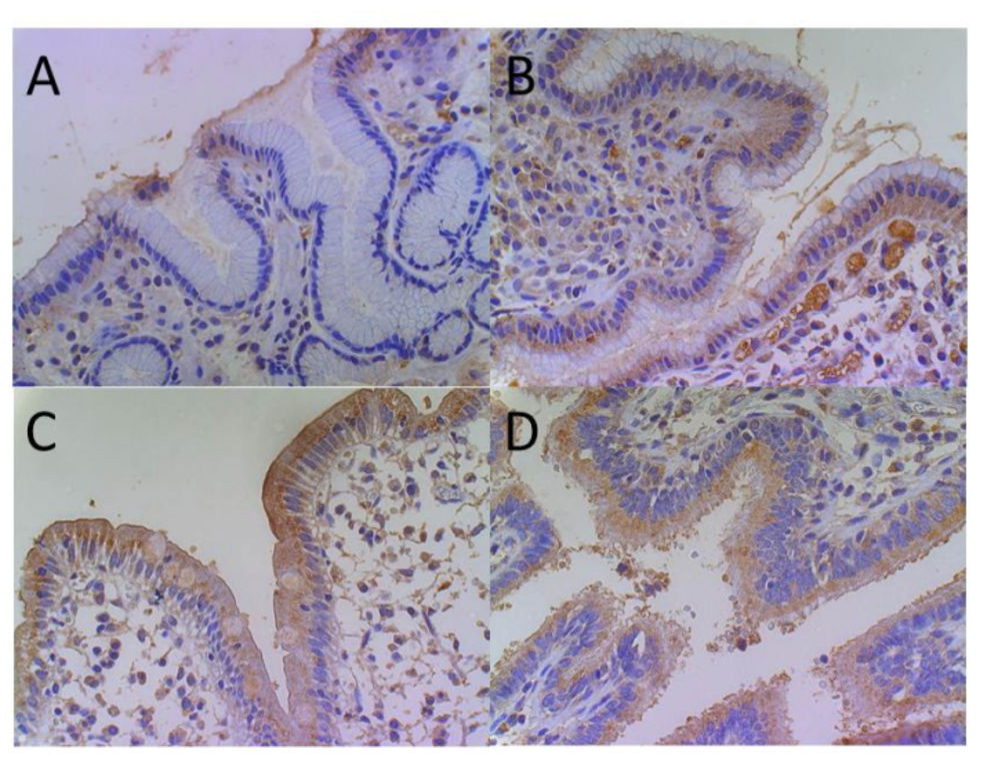
HBD2 immunostaining in *H. pylori* negative and positive mucosal antrum. HBD2 immunohistochemistry shows stronger cytoplasmic brown coloured staining in *H. pylori* positive (B) vs. negative (A) antrum. In Crohn’s colitis (C) and unspecific bronchiolitis (D) HBD2 peptide was also found in epithelial cytoplasm.

**Figure 3:**
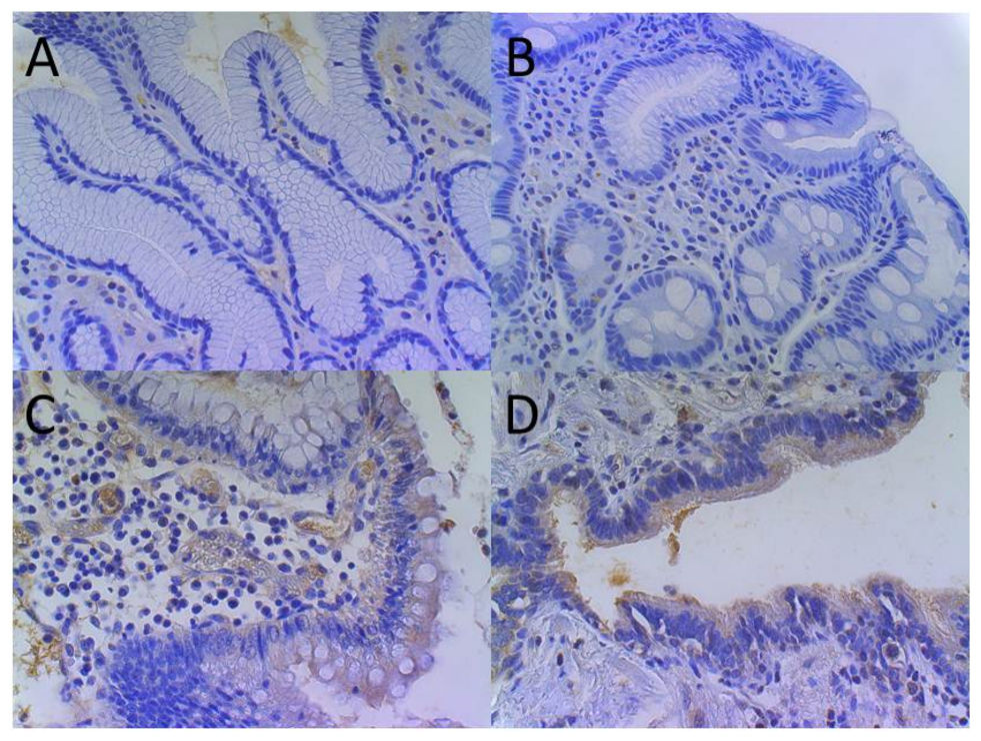
HBD3 immunostaining in *H. pylori* negative and positive mucosal antrum. HBD3 immunohistochemistry shows no or only a marginal staining in both, *H. pylori* negative (A) and positive antrum (B). In contrast, HBD3 is clearly found in the cytoplasm of the epithelium in Crohn’s colitis (C) and unspecific bronchiolitis (D).

**Figure 4:**
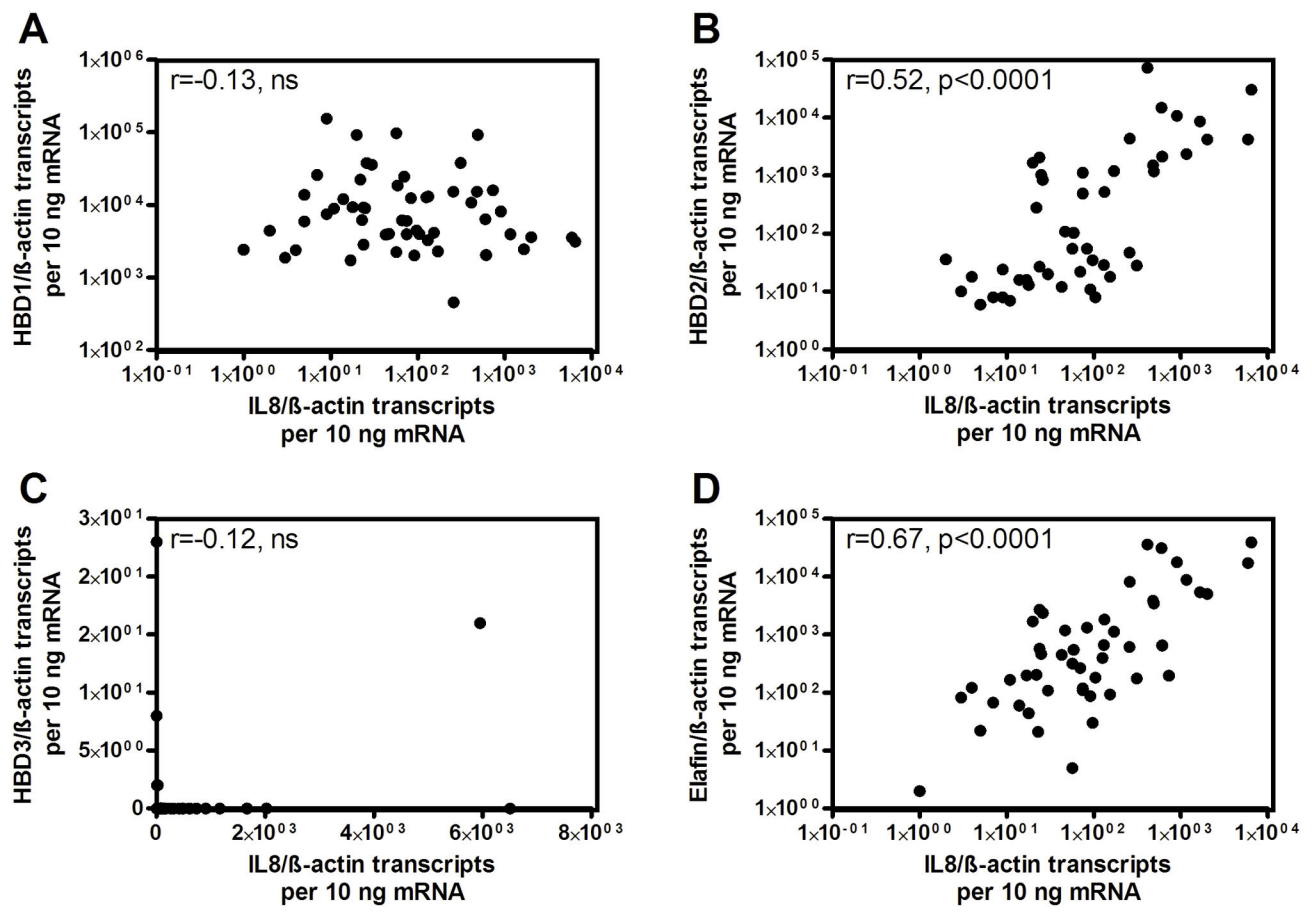
Correlation of IL8 with HBD1, HBD2, HBD3 and elafin. IL8 correlates significantly with HBD2 (B) and elafin (D), but not with HBD1 (A) and HBD3 (C, r = correlation coefficient, ***: p<0.001).

HBD1 (gene name *DEFB1*) transcripts did not differ significantly between *H. pylori* negative and positive patients (Figure 1C). In contrast and concordant with previous reports, HBD2 (gene name *DEFB4A*) and elafin (gene name *PI3*) were significantly induced in *H. pylori* positive mucosa as compared to healthy individuals (antrum: 50-fold for HBD2, p=0.0003 and 18-fold for elafin, p=0.0003; corpus: 105-fold for HBD2, p=0.005 and 37-fold for elafin, p=0.002) (Figure 1 D/G). Accordingly, HBD2 showed a more intense immunostaining in *H. pylori* positive vs. *H. pylori* negative antrum epithelium, with samples of Crohn’s colitis and unspecific bronchiolitis serving as positive controls (Figure 2). Notably, HBD3 (gene name *DEFB103A*) (Figure 1E), HBD4 (gene name *DEFB10A4*) (Figure 1F) and LL37 (gene name *CAMP*) (Figure 1H) transcripts were only marginally detected in human stomach, independent of the *H. pylori* status and without significant differences between the subgroups (less than 50 copies for HBD3 and HBD4 and less than 500 copies for LL37). The minor expression of HBD3 on the mRNA level was also confirmed by immunohistochemistry showing only a weak signal in controls and *H. pylori*-infected patients, whereas HBD3 was clearly positive in the cytoplasm of Crohn’s colitis and unspecific bronchiolitis epithelium (Figure 3). IL8 correlated significantly with HBD2 (correlation coefficient r=0.52, p<0.0001, Figure 4B) and elafin (r=0.67, p<0.0001, Figure 4D), but not with HBD1 (Figure 4A) and HBD3 (Figure 4C). Taken together, HBD1 is constitutively expressed, whereas HBD2 and elafin are induced in *H. pylori* positive antrum and corpus samples by inflammation. In contrast, HBD3, HBD4 and LL37 were barely detected on both mRNA or protein level.

### Bactericidal activity of HBD1-4, LL37 and elafin against *H. pylori* and *E. coli*

**Figure 5:**
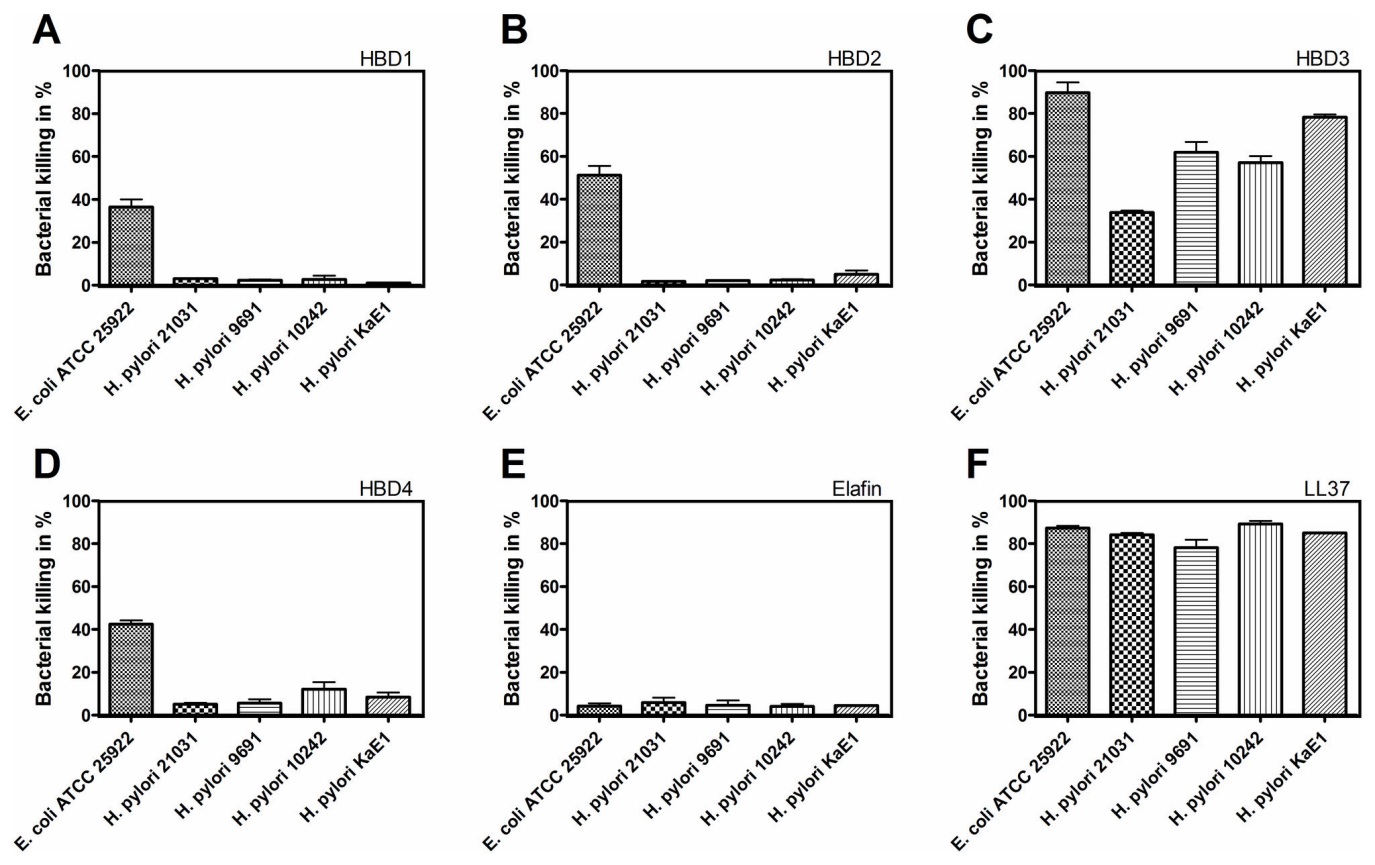
Sensitivity of various *H. pylori* strains to HBD1-4, elafin and LL37. The antibacterial activity of HBD1-4, elafin and LL37 was tested by flow cytometry. Cell damage or cell death lead to the uptake of the green fluorescent dye DiBAC_4_(3). *E. coli* was killed by 20 µg/ml of all recombinant antimicrobials tested. In contrast, *H. pylori* was affected only by HBD3 (C) and LL37 (F) and not by HBD1 (A), HBD2 (B), HBD4 (D) and elafin (E).

Furthermore, we evaluated the antimicrobial effect of recombinant HBD1-4, elafin and LL37 against *E. coli* and *H. pylori* by flow cytometry using a membrane potential sensitive dye. *E. coli* ATCC 25922 was susceptible to HBD1 (37% depolarized bacteria, Figure 5A), HBD2 (51%, Figure 5B) and HBD4 (43%, Figure 5D). In contrast, elafin (Figure 5E) had no bactericidal effect against *E. coli* (4%). HBD3 (Figure 5C) and LL37 (Figure 5F) exhibited the most pronounced bactericidal activity against *E. coli* ATCC 25922 with 90% and 87% depolarized bacterial cells.

With less than 5% depolarized bacteria all 4 *H. pylori* strains were not or only minimally susceptible to the constitutive defensin HBD1, the inducible defensin HBD2 and to elafin (Figure 5A, B and E). Concerning HBD4, *H. pylori* KaE1 and DSM 10242 showed slightly elevated depolarization levels with 8 and 12 %, respectively (Figure 5A, B, D and E). In contrast, HBD3 (Figure 5C) and LL37 (Figure 5F) showed a strong bactericidal effect against *H. pylori* with a range of 34-78% and 62-85% cell depolarization, respectively, among the four strains tested.

**Figure 6:**
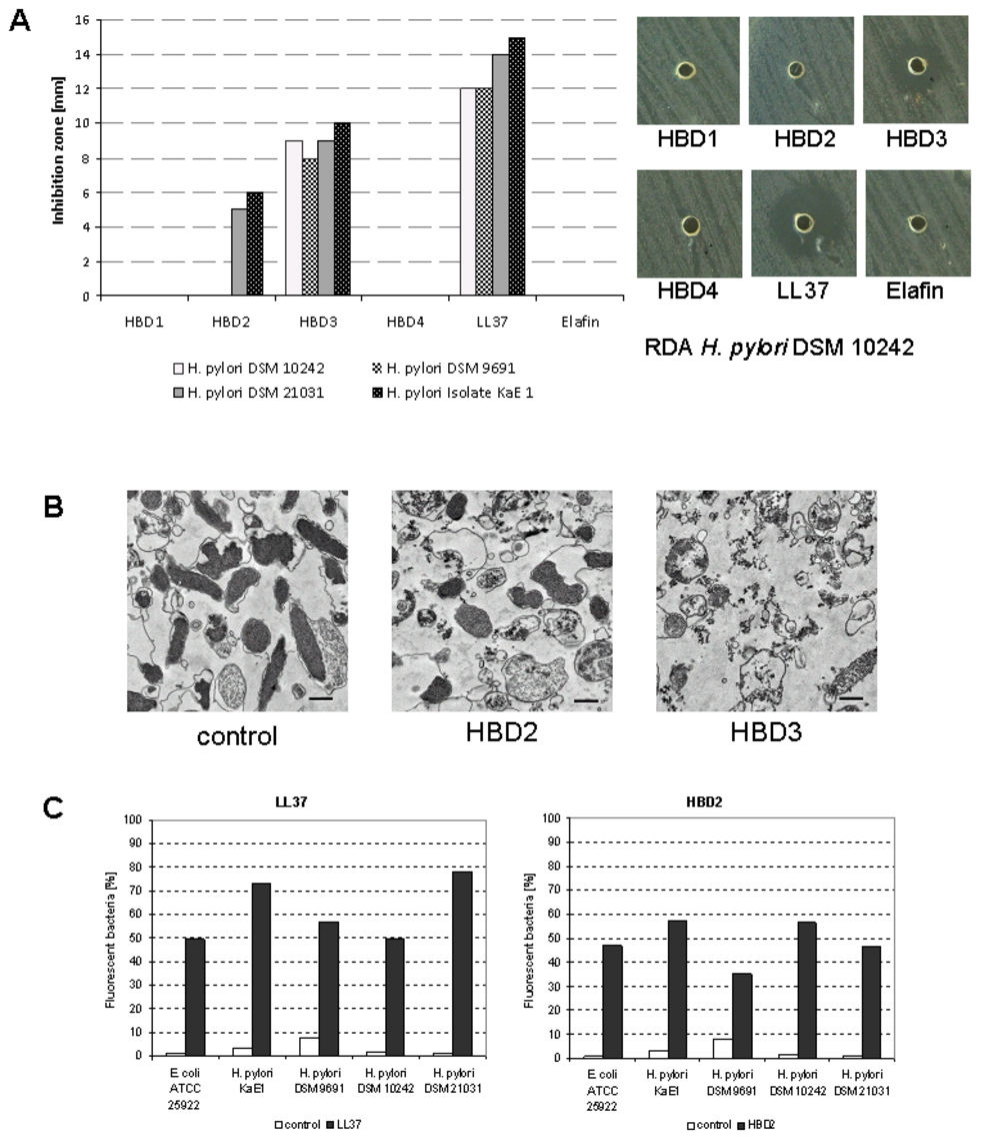
Radial diffusion assay, electron microscopy and binding experiments. In the RDA all four *H. pylori* strains displayed inhibition zones after incubation with 20 µg HBD3 or LL37, but not with HBD1, -4 or elafin. With HBD2 marginal growth inhibition was observed for two strains. Investigated with electron microscopy, cells of *H. pylori* KaE1 showed more pronounced disintegration with 200 µg/ml HBD3 than with 200µg/ml HBD2 (B). In flow cytometric experiments with biotinylated antibodies, HBD2 and LL37 bound to *E. coli* ATCC 25922 and *Helicobacter pylori* (C).

The flow cytometric viability test was confirmed by plating with CFU counting and by radial diffusion assay, measuring inhibition of bacterial growth. CFU were diminished in all Helicobacter strains by HBD3 and LL37, HBD2 inhibited marginally the growth of *H. pylori* KaE 1 (Table 2). In radial diffusion assay we found no zones of inhibition for HBD1, HBD4 and elafin but pronounced zones for LL37 and HBD3 with all four Helicobacter strains (Figure 6A). The activity of HBD2 was strain specific: strains *H. pylori* KaE1 and DSM 21031 formed small inhibition zones, whereas the growth of the two other strains was not influenced by HBD2. In a suspension of *H. pylori* KaE1 investigated with electron microscopy we found much less disintegration of bacterial cells with HBD2 than with HBD3 (Figure 6B). Thus, HBD3 and LL37, the two antimicrobials with negligible gastric expression even during *H. pylori* infection, can potently kill this bacterium, whereas the abundantly expressed antimicrobials HBD1, HBD2 and elafin are only marginally active (HBD2) or inactive (HBD1, elafin) against *H. pylori*. HBD4, that is not expressed in gastric mucosa showed no growth inhibition of *H. pylori*.

**Table 2:** Colony forming units (CFU/ml) of *H. pylori* strains treated with antimicrobial peptides in a concentration of 20 µg/ml (Means of two independent experiments).

	*H. pylori* KaE	*H. pylori* DSM 10242	*H. pylori* DMS 9691	*H. pylori* DSM 21031
Control	1.42 x 10^6^	6.10 x 10^5^	8.85 x 10^5^	7.45 x 10^5^
HBD1	1.32 x 10^6^	6.85 x 10^5^	8.61 x 10^5^	7.15 x 10^5^
HBD2	0.89 x 10^6^	5.92 x 10^5^	7.90 x 10^5^	6.07 x 10^5^
HBD3	0.08 x 10^6^	1.14 x 10^5^	0.20 x 10^5^	1.55 x 10^5^
HBD4	1.26 x 10^6^	4.95 x 10^5^	8.25 x 10^5^	6.90 x 10^5^
LL37	0.09 x 10^6^	0.15 x 10^5^	0.35 x 10^5^	0.41 x 10^5^
Elafin	1.29 x 10^6^	4.85 x 10^5^	9.59 x 10^5^	6.82 x 10^5^

Subsequently we next tested whether HBD2’s lack of killing efficacy was due to inefficient binding to the bacterial membrane. However, binding experiments with HBD2 compared to LL37 showed that both these peptides accumulated on the surface of *E. coli* and *H. pylori* (Figure 6C).

### Antibacterial activity of antrum biopsy extracts in relation to the *H. pylori* status

**Figure 7:**
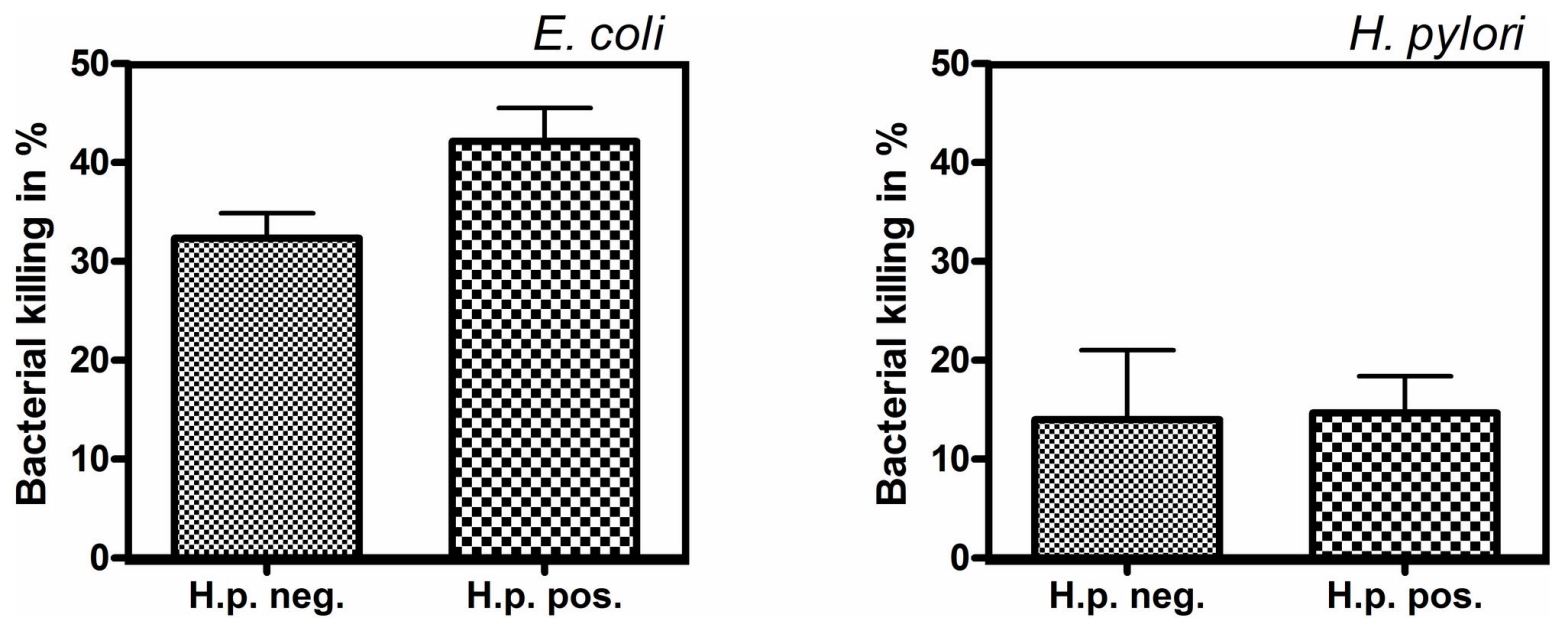
Antimicrobial activity of biopsy extracts. In *H. pylori* positive biopsy extracts antimicrobial activity towards *H. pylori* remained consistent compared with Helicobacter negative extracts. Thus Helicobacter did not induce helicobactericidal factors, whereas in Helicobacter positive extracts the antimicrobial effect towards *E. coli* was increased.

To investigate the impact of antimicrobial peptide induction during Helicobacter infection on the antimicrobial activity of the mucosa we tested 5 *H. pylori* negative and 5 positive antrum biopsy extracts against *E. coli* and *H. pylori*. The incubation of the *H. pylori* negative antrum extracts with *E. coli* led to about 32% depolarized bacteria. In *H. pylori* positive extracts, which showed an induction of several antimicrobial peptides, the bacterial killing of *E. coli* was increased to 42% depolarized cells. In contrast, the antibacterial activity of *H. pylori* negative extracts against *H. pylori* was very low and comparable with the killing in the *H. pylori* positive tissue samples (14% and 15%, Figure 7). Thus, *H. pylori* is not effectively killed by the antibacterial proteins in antrum biopsy extracts in general. Moreover, the induction of antimicrobial peptides such as HBD2 by *H. pylori*, as shown above, did not enhance the killing capacity against *H. pylori*.

## Discussion

The human stomach is protected against microbes by a low gastric pH and by the epithelial secretion of antimicrobial peptides and mucins. Some studies showed a high prevalence of non-*H. pylori* bacteria in the human stomach [1,28], but it remains still unclear, if these bacteria were only transient in healthy subjects or if they invade the gastric mucosa. Hu et al. found a shift in the gastric microbiota in *H. pylori* colonized patients [29]. In healthy subjects without *H. pylori* infection acid resistant microorganisms such as *Veillonella* sp., *Lactobacillus* sp. and *Clostridium* sp. dominate the gastric microbiota [30], whereas *H. pylori* infection leads to other predominant species, namely *Streptococcus* , *Neisseria* , *Rothia* and *Staphylococcus* . Thus, to date, *H. pylori* seems to be the only major and widely distributed pathogen which is able to colonize the bacteria-hostile environment present in the stomach.

There is evidence that *H. pylori* gastritis is associated with an induction of several antimicrobials. For instance, the levels of HBD2 [31,32] and LL37 [20] but not HBD1 [31,32] have been reported to show increased levels in gastric juice of *H. pylori* positive patients. Herein we now compared the expression of all major gastric antibacterial peptides in *H. pylori* positive as compared to non-infected gastric mucosa and related these changes to their anti-*Helicobacter pylori* activity. HBD1 mRNA did not vary significantly between *H. pylori* infected and non-infected gastric mucosa. This is consistent with the majority of previous studies in this field [18,20,33]. Nevertheless, one study also reported an increased [16] and another also a decreased expression [34] of HBD1 in *H. pylori* gastritis. In contrast to HBD1, mRNA and protein expression of HBD2 showed in our study strongly elevated levels in *H. pylori* infected gastric mucosa, similar to previously published data [16–20,33–35].

A surprising finding was the lack of induction of HBD3 in the present study. HBD3 was reported to be induced by *H. pylori* [19,33], whereas in infected children HBD3 was unchanged in gastric antrum and undetectable in corpus biopsies [36]. Both groups measured the mRNA expression in arbitrary units whereas in the present study we determined the total number of HBD3 transcripts. These were quantitated using plasmid standards and exhibited negligibly low levels in gastric *H. pylori* positive and negative samples. In accordance, HBD3 was barely detectable by immunohistochemistry in our *H. pylori* gastritis and control patients. This is in contrast to findings in other inflammatory conditions, such as Crohn’s colitis and bronchiolitis with a strong cytoplasmic staining for HBD3. It is therefore possible that the previously reported relative increase of HBD3 mRNA in *H. pylori* gastritis is rather irrelevant in quantitative terms. The lack of induction of this for *H. pylori* potently fatal peptide is reminiscent of prior findings using *Shigella* species. In this context Islam et al. demonstrated, that turning off LL37 and HBD1 expression allows the pathogen to escape the antimicrobial host defence[37]. . In contrast to Otte et al. [33] and Resnick et al. [38] we found no significant HBD4 expression in the stomach. In a prior work we had also detected HBD4 [39] but we now noted that the primer used in that study amplified HBD2.

The herein presented data furthermore represent the first report of an elafin induction in human stomach by *H. pylori*. Microarray analyses previously showed elafin to be upregulated in *H. pylori* positive rhesus macaques [40] but data based on human samples have so far been missing. The increased levels of elafin mRNA also showed a trend towards the return to normal levels following antibiotic *H. pylori* treatment. LL37 was previously found to be induced in *H. pylori* gastritis [20,33] and eradication resulted in a cessation of this induction [33]. Similar to HBD3, we detected only a small absolute number of LL37 transcripts suggesting that this peptide is quantitatively of minor importance in human stomach.

Notably, it is still unresolved whether *H. pylori* itself [36] and/or the inflammation associated cytokines are responsible for this induction [41]. On the one hand, cell culture experiments performed in different *H. pylori* stimulated gastric adenocarcinoma cell lines (APS, AGS, MKN7, MKN45) showed an increased expression of HBD2 [16,19,42,43], HBD3 [19] and LL37 [20]. On the other hand, stimulation with IL-1β also led to an increase of HBD2 expression [16].

Finally, we tested the susceptibility of three *H. pylori* DSM strains and one clinical isolate towards the most important antimicrobial peptides of mucosal surfaces. In our experiments, the constitutive defensin HBD1 and inducible defensins HBD2 and HBD4 showed a strong antibacterial effect towards *E. coli* but no or only a marginal impact on *H. pylori*. Binding experiments showed that HBD2 accumulated on the cell surface of all four *H. pylori* strains tested even though it seemed to exert no major negative effects on the bacterium’s viability. In the radial diffusion assay a marginal inhibitory effect of HBD2 on bacterial growth was demonstrated to be strain specific: only growth of *H. pylori* KaE1 and *H. pylori* 21031 was slightly inhibited by HBD2. Even though KaE1 electron microscopy revealed a deleterious effect by HBD2 on bacterial membrane structure, the flow cytometric assay demonstrated no significant depolarisation of bacterial cells. In contrast, with HBD3 and LL37 we achieved clear zones of inhibition for all strains tested and bacteria-free areola were always greatly exceeding those generated by HBD2 in two of four strains. Thus, the binding of HBD2 to the bacteria resulted in limited structural changes but no effective killing and only strain specific, minor growth inhibition of *H. pylori*. Possibly, as recently shown by Cullen et al. the modification of lipid A by removing phosphate groups, which results in a comparably less negative surface charge, increases the resistance of *H. pylori* to LL37 and HBD2 [44].

Previously, Hase et al. [20] had tested two *H. pylori* strains against the antimicrobials HBD1, HBD2 and LL37 and for HBD2, one strain tested was resistant to HBD1, while the second strain was sensitive. On the other hand, George et al. detected with 10^-7^ M recombinant HBD1 in hypotonic buffer a reduction of CFU of about 50% and an absolute eradication with 10^-5^ M [21]. In the study by Hase et al. [20] HBD2 had no effect on bacterial viability whereas in a study by Kawauchi et al. 30 µg/ml HBD2 caused growth inhibition of 70% and a complete inhibition was achieved at 50 µg/ml [19]. The different findings may result from the strain specific effect of HBD2 which we observed as well as methodical variability regarding molarity and buffers. With respect to HBD3 George et al. also showed that this defensin is most effective against *H. pylori*, but as we now report, this peptide may well be absent from gastric mucus [21]. Similarly, we confirmed an inhibitory activity of LL37 but its expression also appears to be negligible. For elafin, antimicrobial activity is described for *Pseudomonas aeruginosa* and *Staphylococcus aureus*, but not for *E. coli* [9,45]. We observed no bactericidal effect against *E. coli* or *H. pylori* with elafin peptides from different companies but we also observed only a limited killing of *P. aeruginosa* (data not shown).

In general, in our study the cag- and vac-positive strain *H. pylori* DSM 10242 and the three virulence factor-negative strains did not differ in the susceptibility towards antimicrobial peptides. Taken together, we conclude that only HBD3 and LL37 were consistently potent against all strains of *H. pylori*. Since they are produced in low quantity in the stomach, if at all, these antimicrobial peptides fail to eradicate *H. pylori*. Furthermore biopsy extracts from the antrum with and without infection were tested for antimicrobial activity and showed little effect on *H. pylori* in contrast to the bactericidal effect on *E. coli*, that was stronger in extracts of infected biopsies.

In conclusion, our results suggest that *H. pylori* has developed resistance mechanisms against constitutive antimicrobial host factors, such as HBD1, and even factors induced by Helicobacter infection such as HBD2. It may be speculated that the induction of HBD2 contributes to minimize competition by other, more susceptible bacteria. In addition, Helicobacter does not induce potentially helicobactericidal peptides like HBD3 and LL37. The combination of selective defensin induction and resistance to others may enable Helicobacter to colonize the gastric mucus layer where it can adhere to epithelial cells and induce inflammatory as well as malignant processes. A better understanding of the mechanisms regarding *H. pylori* selective antimicrobial resistance and susceptibility against different peptides might help to identify potential targets for novel eradication therapeutics.

## Supporting Information

Table S1 Sequences of recombinant peptides used in the antimicrobial assays.
(DOC)
