# Supplementary material for: Gastric Antimicrobial Peptides Fail to Eradicate Helicobacter pylori Infection Due to Selective Induction and Resistance
Source: PLoS One. 2013 Sep 11;8(9):e73867. doi: 10.1371/journal.pone.0073867 (PMC3770654; doi:10.1371/journal.pone.0073867)
Supplement: Table S1 — Sequences of recombinant peptides used in the antimicrobial assays. (DOC) [file pone.0073867.s001.doc]

**Gastric antimicrobial peptides fail to eradicate *Helicobacter pylori* infection due to selective induction and resistance**

Sabine Nuding^1^*; Michael Gersemann^2^*; Yoshio Hosaka^3^; Sabrina Konietzny^2^; Christian Schaefer^2^; Julia Beisner^1^; Bjoern O. Schroeder^1^; Maureen J. Ostaff^1^; Katunori Saigenji^4^; German Ott^5^; Martin Schaller^6^; Eduard F. Stange^2^; Jan Wehkamp^1,2^

^1^Dr. Margarete Fischer-Bosch-Institute of Clinical Pharmacology, Stuttgart, and University of Tübingen, Germany

^2^Department of Internal Medicine I, Robert-Bosch Hospital, Stuttgart, Germany

^3^Department of Internal Medicine, ISUZU Hospital, Tokyo, Japan

^4^Department of Gastroenterology, Kitasato University, Kanagawa, Japan

^5^Department of Clinical Pathology, Robert-Bosch Hospital, Stuttgart, Germany

^6^Department of Dermatology, Eberhard Karls University Tübingen, Tübingen, Germany

**Supporting information**

**Table S1:** Sequences of each recombinant peptide used in the antimicrobial assays

| **Antimicrobial Peptide** | **Sequence** |
| --- | --- |
| **HBD1** | Asp - His - Tyr - Asn - Cys - Val - Ser - Ser - Gly - Gly - Gln - Cys - Leu - Tyr - Ser - Ala - Cys - Pro - Ile - Phe - Thr - Lys - Ile - Gln - Gly - Thr - Cys - Tyr - Arg - Gly - Lys - Ala - Lys - Cys - Cys - Lys |
| **HBD2** | Gly - Ile - Gly - Asp - Pro - Val - Thr - Cys - Leu - Lys - Ser - Gly - Ala - Ile - Cys - His - Pro - Val - Phe - Cys - Pro - Arg - Arg - Tyr - Lys - Gln - Ile - Gly - Thr - Cys - Gly - Leu - Pro - Gly - Thr - Lys - Cys - Cys - Lys - Lys - Pro |
| **HBD3** | Gly - Ile - Ile - Asn - Thr - Leu - Gln - Lys - Tyr - Tyr - Cys - Arg - Val - Arg - Gly - Gly - Arg - Cys - Ala - Val - Leu - Ser - Cys - Leu - Pro - Lys - Glu - Glu - Gln - Ile - Gly - Lys - Cys - Ser - Thr - Arg - Gly - Arg - Lys - Cys - Cys - Arg - Arg - Lys - Lys |
| **HBD4** | Glu - Leu - Asp - Arg - Ile - Cys - Gly - Tyr- Gly - Thr - Ala - Arg - Cys - Arg - Lys - Lys - Cys - Arg - Ser - Gln - Glu - Tyr - Arg - Ile - Gly - Arg - Cys - Pro - Asn - Thr - Tyr - Ala - Cys - Cys - Leu - Arg - Lys |
| **LL37** | Leu - Leu - Gly - Asp - Phe - Phe - Arg - Lys - Ser - Lys - Glu - Lys - Ile - Gly - Lys - Glu - Phe - Lys - Arg - Ile - Val - Gln - Arg - Ile - Lys - Asp - Phe - Leu - Arg - Asn - Leu - Val - Pro - Arg - Thr - Glu - Ser |
| **Elafin** | Ala - Gln - Glu - Pro - Val - Lys - Gly - Pro - Val - Ser - Thr - Lys - Pro - Gly - Ser - Cys - Pro - Ile - Ile - Leu - Ile - Arg - Cys - Ala - Met - Leu - Asn - Pro - Pro - Asn - Arg - Cys - Leu - Lys - Asp - Thr - Asp - Cys - Pro - Gly - Ile - Lys - Lys - Cys - Cys - Glu - Gly - Ser - Cys - Gly - Met - Ala - Cys - Phe - Val - Pro - Gln |
